# Integrating network pharmacology and molecular docking to explore the pharmacological mechanism of tanshinone IIA in improving chronic obstructive pulmonary disease

**DOI:** 10.1097/MD.0000000000041638

**Published:** 2025-03-21

**Authors:** Huaiquan Liu, Shili Yang, Bo Chen, Shuoshuo Shao, Xinyan Zhang

**Affiliations:** a Guizhou University of Traditional Chinese Medicine, Guiyang, China.

**Keywords:** COPD, molecular docking, network pharmacology, tanshinone IIA

## Abstract

This study explores the mechanism of action of tanshinone IIA in the treatment of chronic obstructive pulmonary disease (COPD) using network pharmacology and molecular docking. The targets of tanshinone IIA were searched by Swiss Target Prediction Database, PharmMapper Database, SuperPred Database, and TargetNet Database. The targets of COPD were obtained by Genecards Database, OMIM Database, and Therapeutic Target Database, then the intersection targets were selected as the targets of tanshinone IIA in the treatment of COPD. The intersecting targets were imported into the STRING database to obtain the PPI network and the top10 relevant targets, and GO enrichment and KEGG signaling pathway analysis were performed by R language. Core targets were obtained by taking the intersection of Top5 GO and KEGG corresponding targets with Top10 targets in PPI. Then tanshinone IIA was molecularly docked to the screened core target protein receptors by AutoDock Vina software. Tanshinone IIA included 442 potential targets and 979 COPD-associated targets, and 104 intersecting targets were obtained by taking the intersection of the two. The PPI network showed that ALB, EGFR, CASP3, MMP9, PTGS2, NFKB1, ESR1, SRC, PPARG, and HSP90AA1 were the top 10 relevant targets. GO enrichment analyses showed that the main components involved were the response to response to lipopolysaccharide, response to molecule of bacterial origin, positive regulation of cytokine production, positive regulation of MAPK cascade, and positive regulation of kinase activity. KEGG signaling pathway analysis revealed major involvement in prostate cancer, AGE-RAGE signaling pathway in diabetic complications, Hepatitis B, PI3K-Akt signaling pathway, relaxin signaling pathway. EGFR, CASP3, MMP9, NFKB1, SRC, and HSP90AA1 were the 6 core targets. Molecular docking showed that the binding energies of tanshinone IIA and the core target were all less than ≤−5.0 kcal/mol, demonstrating good affinity. The treatment of COPD with tanshinone IIA involves multiple signaling pathways and biological processes, and its binding to the key targets of EGFR, CASP3, MMP9, NFKB1, SRC, and HSP90AA1 may be one of the important mechanisms of its action, which provides new theoretical ideas for the subsequent treatment of COPD with tanshinone IIA.

## 1. Introduction

Chronic obstructive pulmonary disease (COPD) is a heterogeneous lung condition characterized by progressive and incompletely reversible airflow limitation, with clinical manifestations of chronic respiratory symptoms such as cough, sputum, dyspnea and respiratory abnormalities such as chronic bronchitis, bronchiectasis and emphysema.^[1]^ Epidemiological studies have shown that COPD has become the third largest mortality factor in the world, affecting about 300 million people globally, and is also characterized by high prevalence, high mortality and high disability, affecting 64 million disability-adjusted life years, and bringing a heavy economic burden to both patients and society.^[2]^ The pathogenesis of COPD is closely related to factors such as smoking, occupational dust, chemicals, air pollution, infectious agents, and immune dysfunction, but its pathogenesis is unclear and involves mainly mechanisms such as inflammatory response, oxidative stress, and protease-antiprotease imbalance. Currently, β-agonists and anticholinergic drugs, glucocorticoids, expectorants and antibiotics are often used in the clinic to alleviate the symptoms of coughing up phlegm, shortness of breath, wheezing, chest tightness or dyspnea in COPD patients, but there are certain side effects.^[3]^ Therefore, there is an urgent need to develop new therapeutic strategies for the treatment of COPD with new drugs and disease targets.

Traditional Chinese medicine and natural products are the best source of active ingredients for drug discovery, favoring the treatment of diseases. Tanshinone IIA is a fat-soluble ingredient isolated from the Traditional Chinese medicine Danshen, and sodium tanshinone IIA sulfonate (STS) is its sodium salt, which still plays a role in the composition of tanshinone IIA.^[4]^ Modern pharmacological studies have shown that tanshinone IIA has a wide range of pharmacological effects, not only improving cardiovascular diseases by improving microcirculation and dilating blood vessels, but also preventing and controlling respiratory diseases through antioxidant, immune cell regulation and anti-inflammatory effects.^[5,6]^ Tanshinone IIA improved tidal volume, breaths per minute and arterial blood gas levels in a lipopolysaccharide (LPS)-induced acute lung injury model in rats through antioxidant and anti-inflammatory effects.^[7]^ Tanshinone IIA could regulate macrophage polarization, reducing the expression of M1 macrophages and increasing the expression of M2 macrophages, to improve lung conditions in an LPS-induced acute lung injury mouse.^[8]^ Both tanshinone IIA and STS could improve lung function impairment in cigarette smoke and LPS-induced COPD mice by regulating miR-486-5p.^[9]^ In hypoxic conditions, human lung epithelial cells (A549 cells) pretreated with tanshinone IIA could enhance cell survival by upregulating the expression of genes such as iNOS, HO-1, jun-D, c-jun, and fos B through the Nrf2-AP-1 pathway.^[10]^ Based on the above findings, tanshinone IIA were considered a potential therapeutic drug for respiratory system diseases, particularly in the treatment of COPD. However, the underlying mechanism of its action in treating COPD remains unclear. Therefore, this study will utilize network pharmacology and molecular docking to investigate the mechanism of action of tanshinone IIA in the treatment of COPD.

## 2. Materials and methods

### 2.1. Collection and screening of potential targets of tanshinone IIA

The PubChem database (https://pubchem.ncbi.nlm.nih.gov/) was used to obtain the Canonical SMILES number for tanshinone IIA. Then, the resulting number was entered into the SwissTargetPrediction database (http://www.swisstargetprediction.ch/index.php), the PharmMapper database (http://www.lilab-ecust.cn/pharmmapper/), the SuperPred database (https://prediction.charite.de/), SuperPred database (http://targetnet.scbdd.com/), and TargetNet database (http://targetnet.scbdd.com/) to obtain targets. Finally, Standardize the species as “Homo sapiens” in the UniProt database for further processing.

### 2.2. Collection and processing of COPD targets

The GeneCards database (https://www.genecards.org/), OMIM database (https://omim.org/) and Therapeutic Target Database database (https://db.idrblab.net/ttd/) were screened for COPD-related genes using the keyword “chronic obstructive pulmonary disease.” The GeneCards database was set to a relevance score of ≥ 30, and the 3 databases were integrated to obtain relevant targets for COPD.

### 2.3. Constructing a protein-protein interaction (PPI) network of target proteins

Using the “VennDiagram” package in R, the intersection of tanshinone IIA target genes and COPD-related target genes were determined to identify potential targets for tanshinone IIA in the treatment of COPD. The intersecting targets were uploaded into the STRING database (https://cn.string-db.org/), and the species was selected as “Homo sapiens,” and the confidence level was set to be greater than 0.4 to construct a PPI network for the targets. In the PPI network, nodes represent target proteins and edges represent interactions between target proteins. Degree centrality represents the number of connections between a node and other nodes in the PPI network. The higher degree value indicates the higher importance of the node in the network, and the PPI network graph was drawn by Cytoscape 3.10.0 software.

### 2.4. Gene ontology (GO) enrichment and kyoto encyclopedia of genes and genomes (KEGG) pathway analysis

Gene symbols were mapped to Entrez Gene IDs by the R package “org.Hs.e.g..db,” and GO enrichment and KEGG pathway analysis were performed by the package “clusterProfiler.” The top 20 KEGG pathway and top 10 GO enrichment were screened to plot and bubbles using *P*.adjust <.05 as the screening criterion. Then, the genes corresponding to the top 5 in the KEGG pathway and GO enrichment were plotted sankey map. Finally, the intersection of the targets corresponding to the top 5 entries in the KEGG pathway and GO enrichment with the top 10 targets in the PPI were taken to obtain the core targets, and the venn diagram and PPI network map were drawn.

### 2.5. Molecular docking

The core targets EGFR (PDB ID:7U99), CASP3 (PDB ID:1RE1), MMP9 (PDB ID:6ESM), NFKB1 (PDB ID:1SVC), SRC (PDB ID:1O41), and HSP90AA1 (PDB ID:3O0I) were molecularly docked against tanshinone IIA. Firstly, the 2D and 3D structures of tanshinone IIA were searched using the PubChem database and saved in SDF format, and then the 3D structure was optimized using ChemBioOffice software and the SDF format was converted to mol2 format.

The protein structures of the core targets were searched in the RCSB PDB database (https://www.rcsb.org/) and the receptor proteins were made to remove water molecules and add hydrogen atoms using the AutoDock Tools software. Molecular docking was performed with Autodock Vina software and the minimum binding energy was calculated and 3D plots of molecular docking results were generated by PyMol software and 2D plots of molecular docking results were drawn by LigPlot software.

## 3. Results

### 3.1. Potential targets of tanshinone IIA

**Figure 1.**
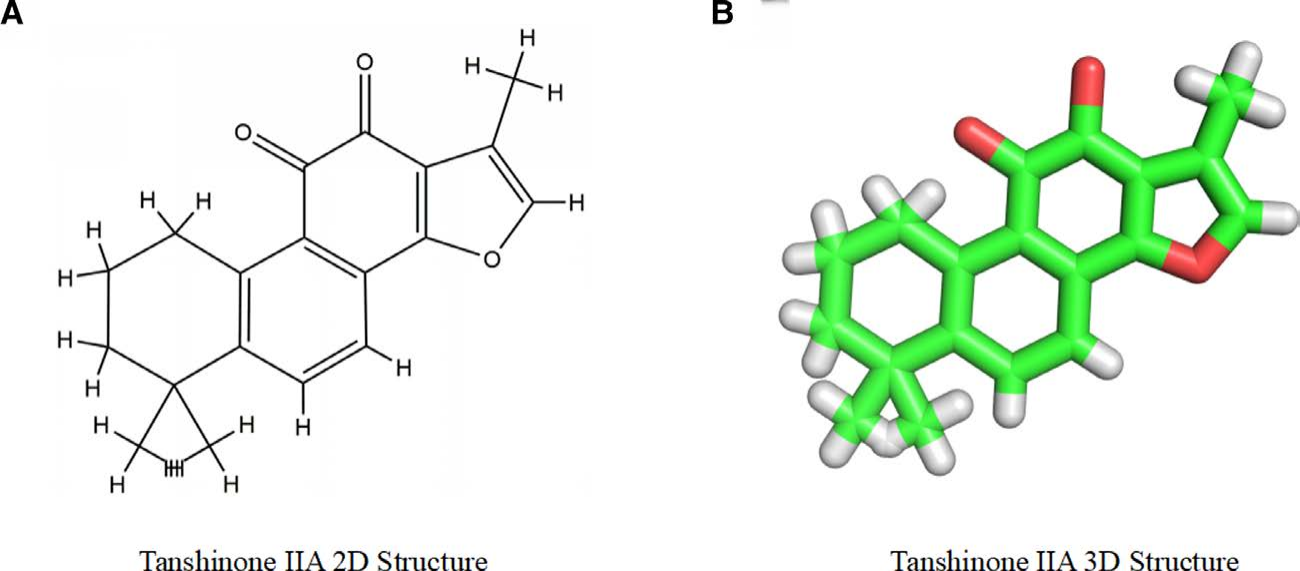
2D and 3D structures of tanshinone IIA.

Parameters of tanshinone IIA obtained from the PubChem database are presented in Table 1, while the 2D and 3D structures are shown in Figure 1. The SwissTargetPrediction database yielded 46 targets, the PharmMapper database yielded 241 targets, the SuperPred database yielded 146 targets, the TargetNet database yielded 93 targets, and the removal of duplicates resulted in 442 potential targets.

**Table 1:** Parameters of tanshinone IIA.

Name	PubChem CID	Molecular formula	Molecular weight	CAS	Canonical SMILES
Tanshinone IIA	164676	C19H18O3	294.3 g/mol	568-72-9	CC1 = COC2 = C1C(=O)C(=O)C3 = C2C = CC4 = C3CCCC4(C)C

### 3.2. Targets of COPD

**Figure 2.**
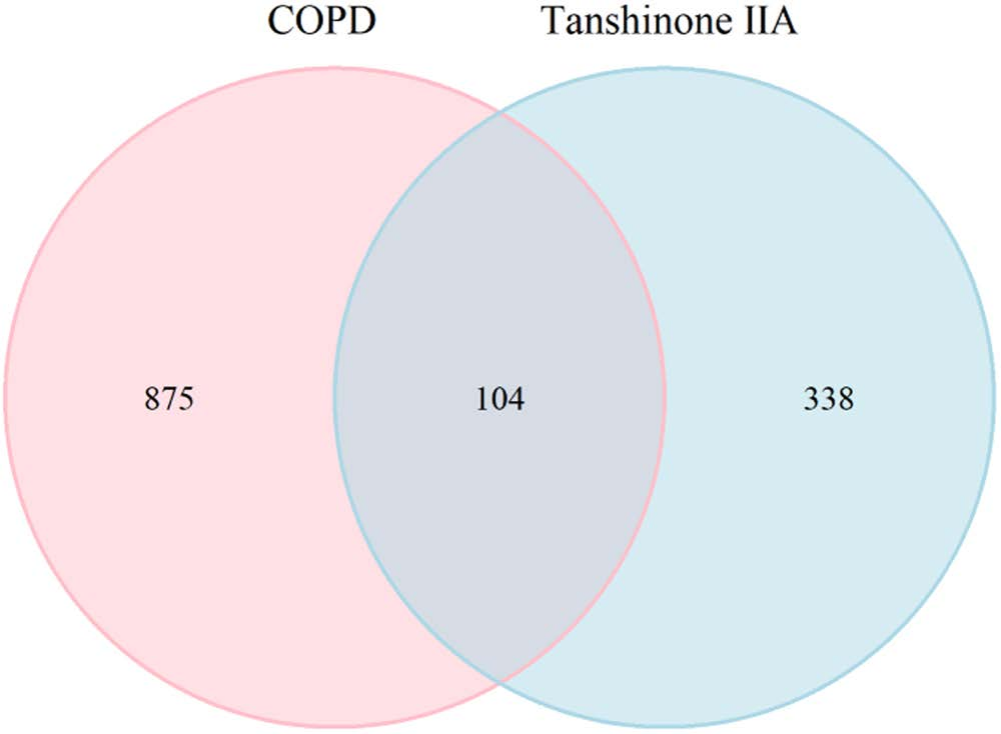
Intersecting targets of tanshinone IIA and COPD. COPD = chronic obstructive pulmonary disease.

808 targets were obtained in Genecards database after using Relevance score ≥ 30 as a parameter, 200 targets were obtained in OMIM database, 66 targets were obtained in Therapeutic Target Database database, and 979 relevant targets were obtained by removing duplicates. 104 intersecting targets were obtained by taking the 442 potential targets of tanshinone IIA and 979 targets of COPD, as shown in Figure 2.

### 3.3. PPI network

**Figure 3.**
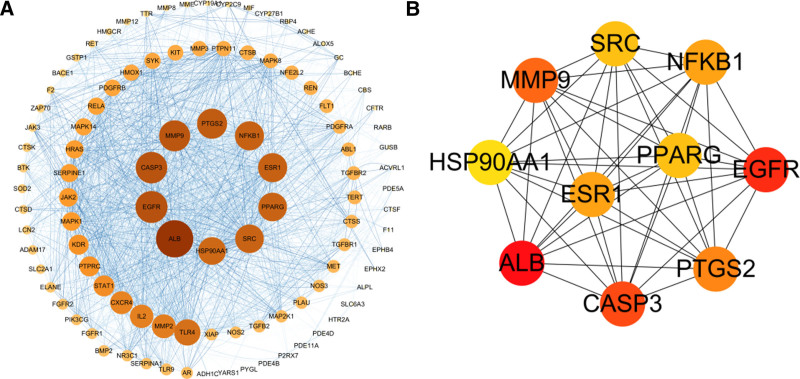
PPI network of tanshinone IIA treating COPD targets. COPD = chronic obstructive pulmonary disease.

In the STRING database, a protein-protein interaction (PPI) network of tanshinone IIA treating COPD was obtained, and further visualized using Cytoscape 3.10.0 software. The network consists of 103 targets (GM2A targets were excluded from interacting with other targets) and 2616 interaction edges. Larger and redder nodes indicate higher degree values for corresponding targets, while bluer edges represent higher association strength, as shown in Figure 3A. Using the cytoHubba plugin, the top 10 targets based on degree values were ALB, EGFR, CASP3, MMP9, PTGS2, NFKB1, ESR1, SRC, PPARG, and HSP90AA1, as shown in Figure 3B.

### 3.4. GO enrichment and KEGG pathway analysis

**Figure 4.**
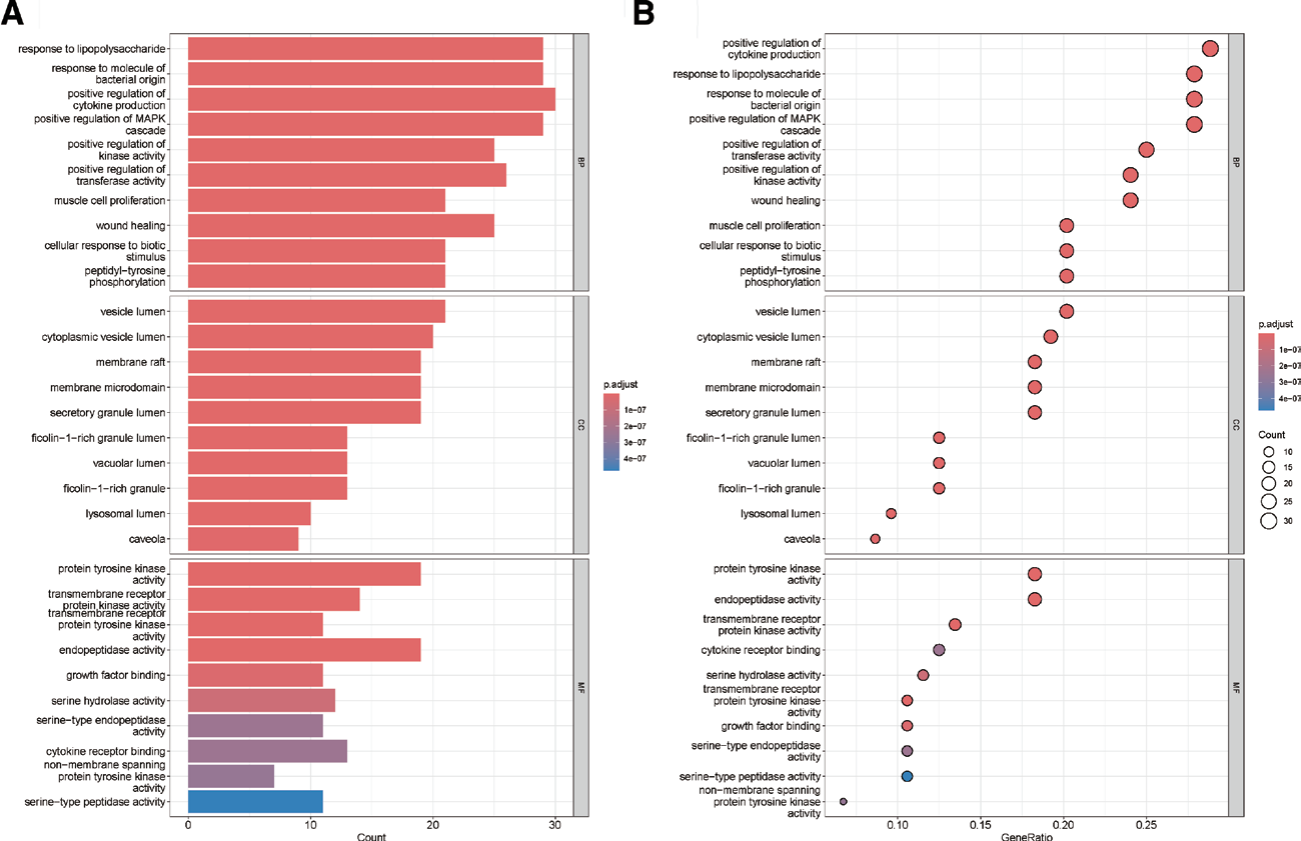
Bar plot and bubble plot of the top 10 GO enrichment. GO = gene ontology.

**Figure 5.**
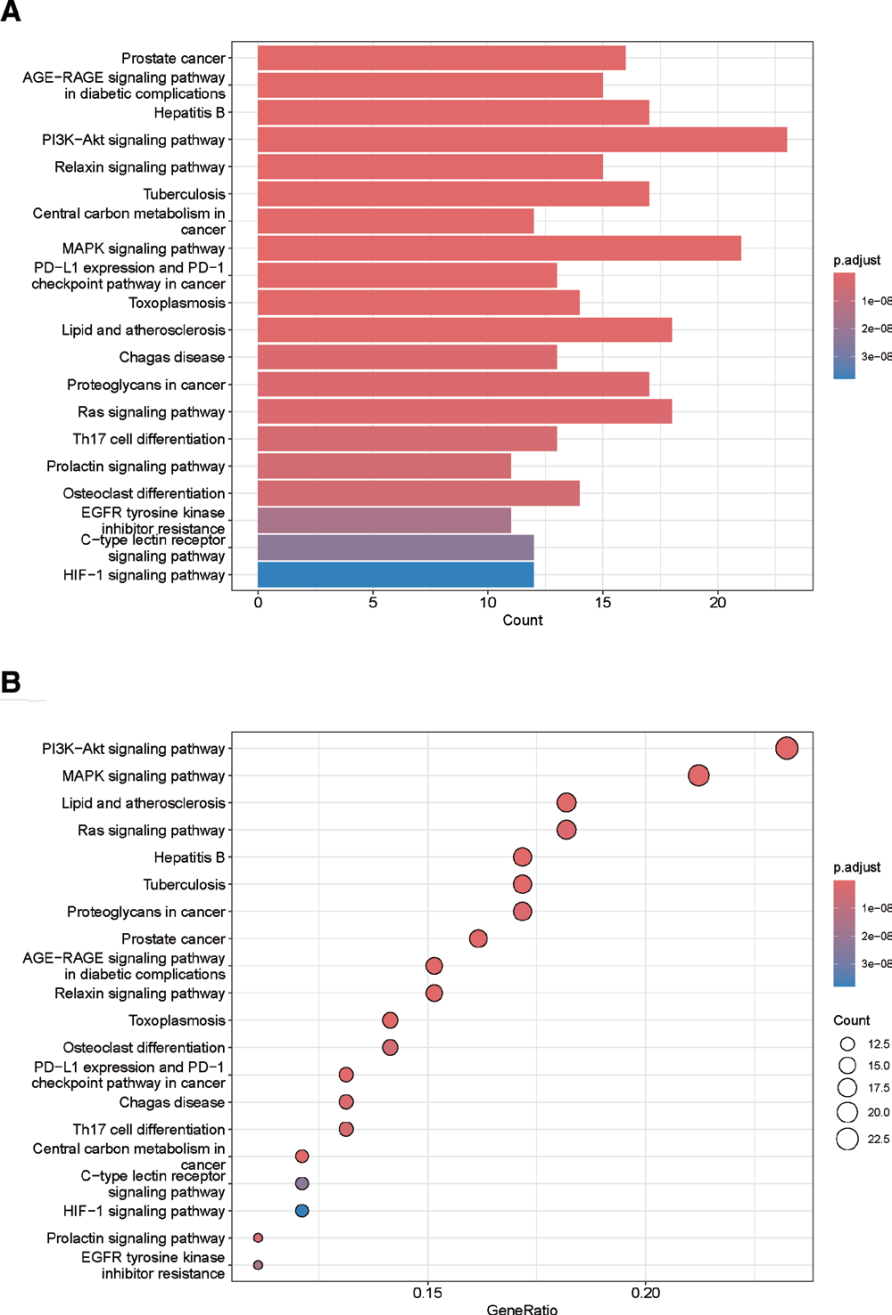
Bar plot and bubble plot of the top 20 KEGG pathways. KEGG = Kyoto Encyclopedia of Genes and Genomes.

**Figure 6.**
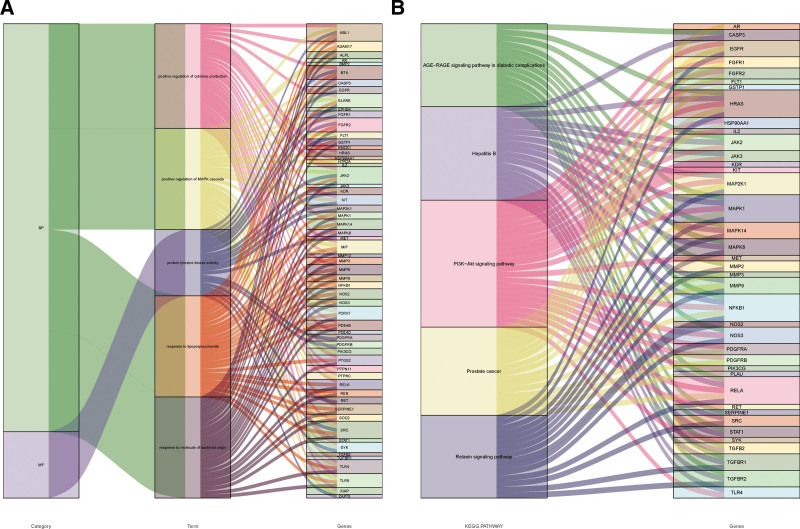
Sankey diagram of the top 5 corresponding targets between GO enrichment and KEGG pathways. GO = gene ontology, KEGG = Kyoto Encyclopedia of Genes and Genomes.

**Figure 7.**
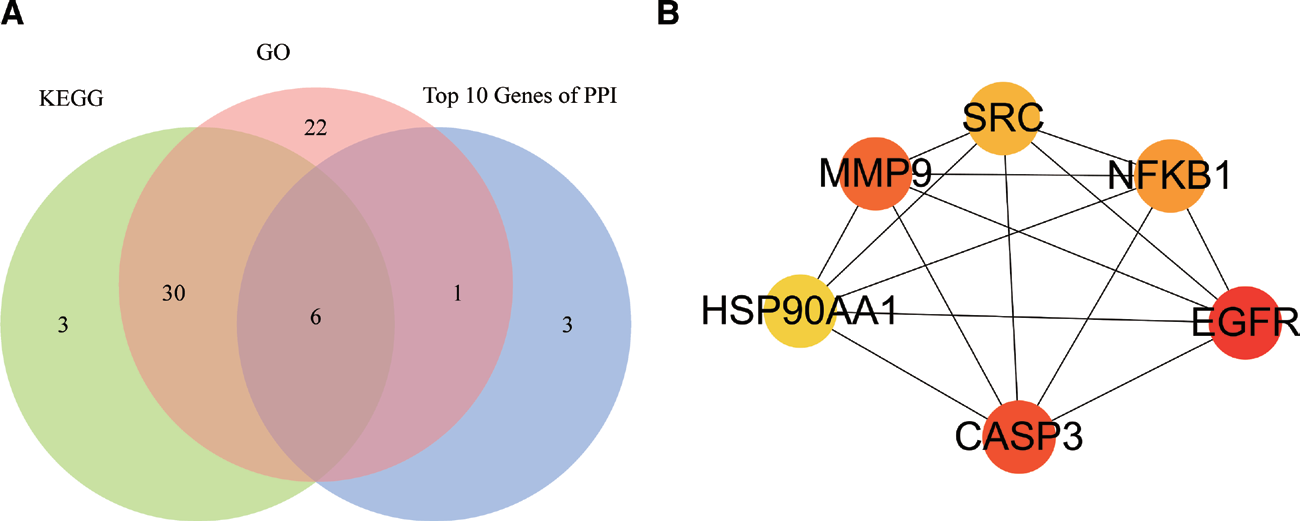
Venn diagram and PPI network of core targets.

GO enrichment and KEGG pathway analyses were performed on 104 intersecting targets. There were 1781 entries for biological process, 52 entries for cellular component and 139 entries for molecular function in the GO enrichment, mainly involving positive regulation of response to lipopolysaccharide, positive regulation of MAPK cascade and positive regulation of kinase activity. These enrichments were related to response to lipopolysaccharide, response to molecule of bacterial origin, positive regulation of cytokine production, positive regulation of MAPK cascade, positive regulation of kinase activity, as shown in Figure 4. KEGG pathways analyses had 133 pathways, and the top ones were mainly involved inProstate cancer, AGE-RAGE signaling pathway in diabetic complications, Hepatitis B, PI3K-Akt signaling pathway, Relaxin signaling pathway, as shown in Figure 5. The GO enrichment and KEGG pathways of TOP5 mapped 59 and 39 targets, respectively, as shown in Figure 6. The intersection of the top 5 GO enrichment and KEGG pathway targets with the top 10 genes in the PPI network resulted in 6 core targets: EGFR, CASP3, MMP9, NFKB1, SRC, and HSP90AA1, as shown in Figure 7.

### 3.5. Molecular docking

**Figure 8.**
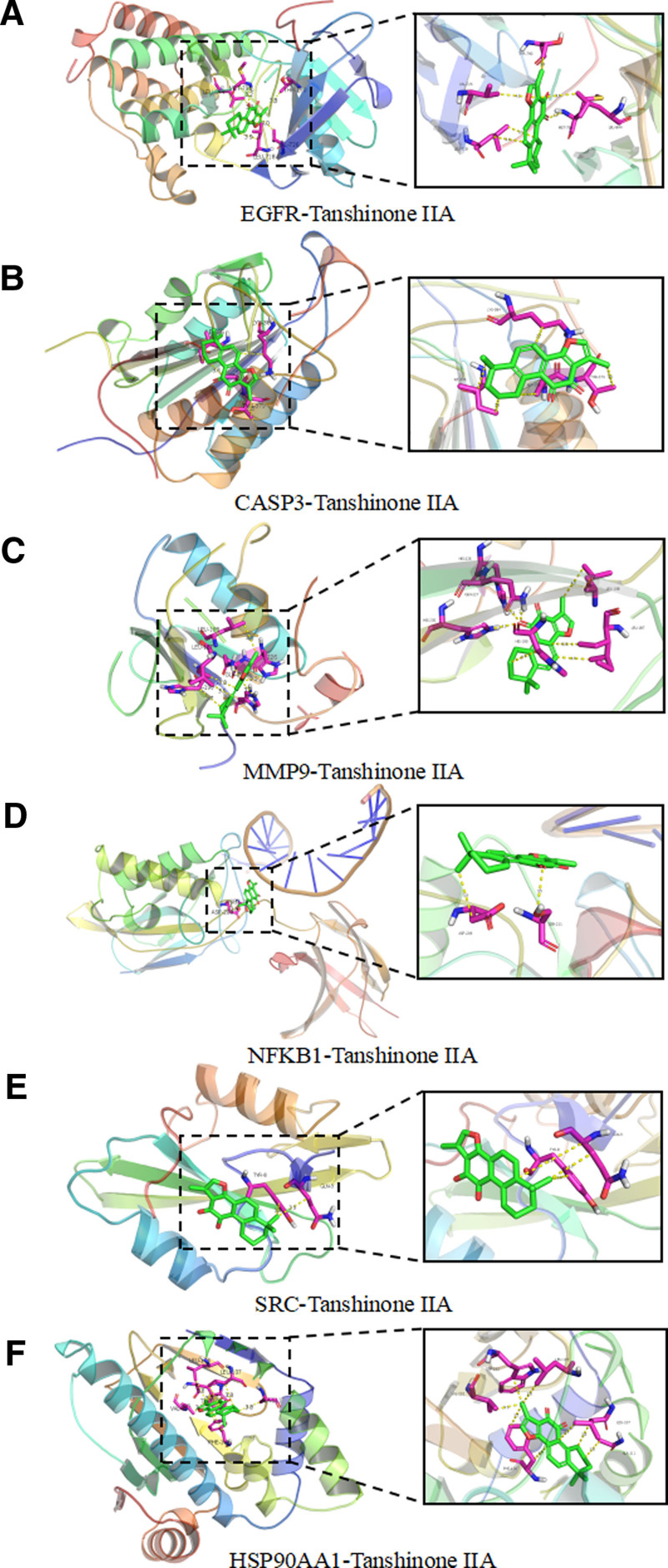
Cartoon representation of the docking of Tanshinone IIA with protein receptors.

**Figure 9.**
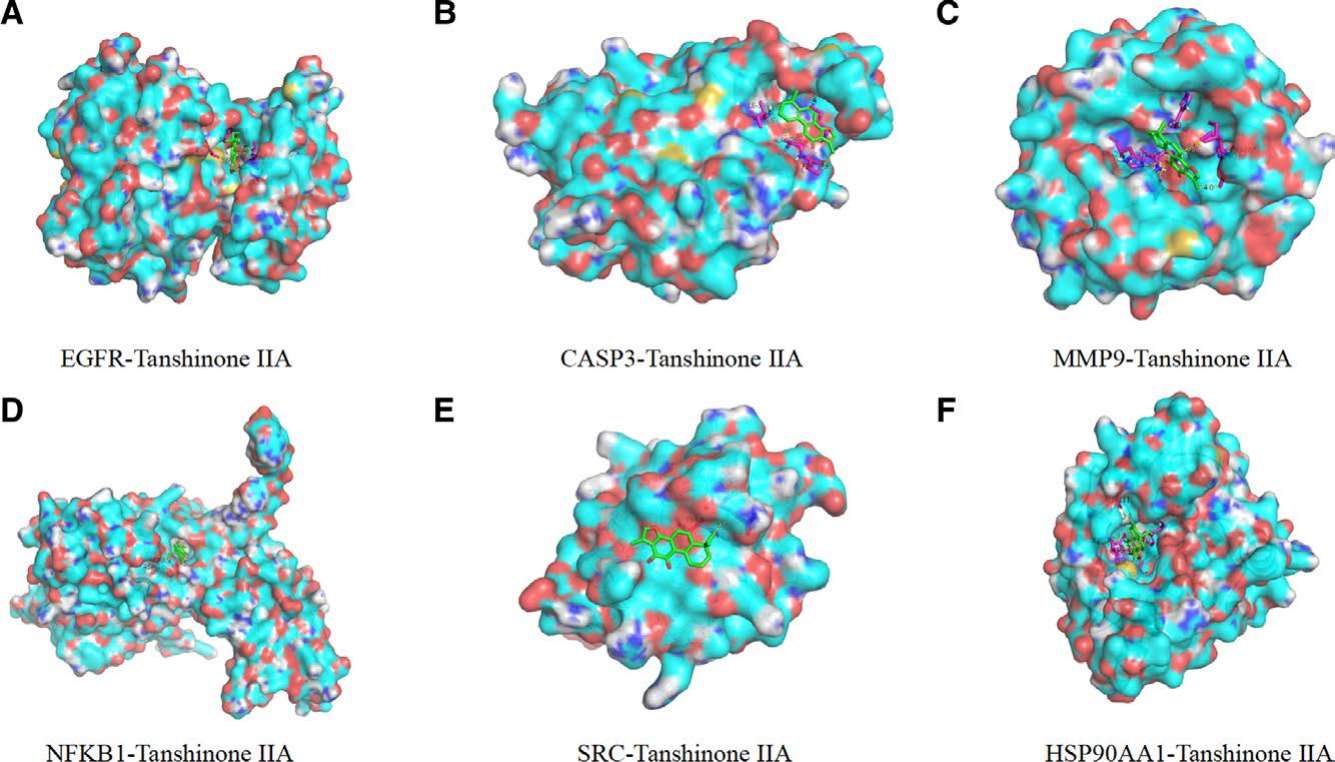
Surface representation of the docking of Tanshinone IIA with protein receptors.

**Figure 10.**
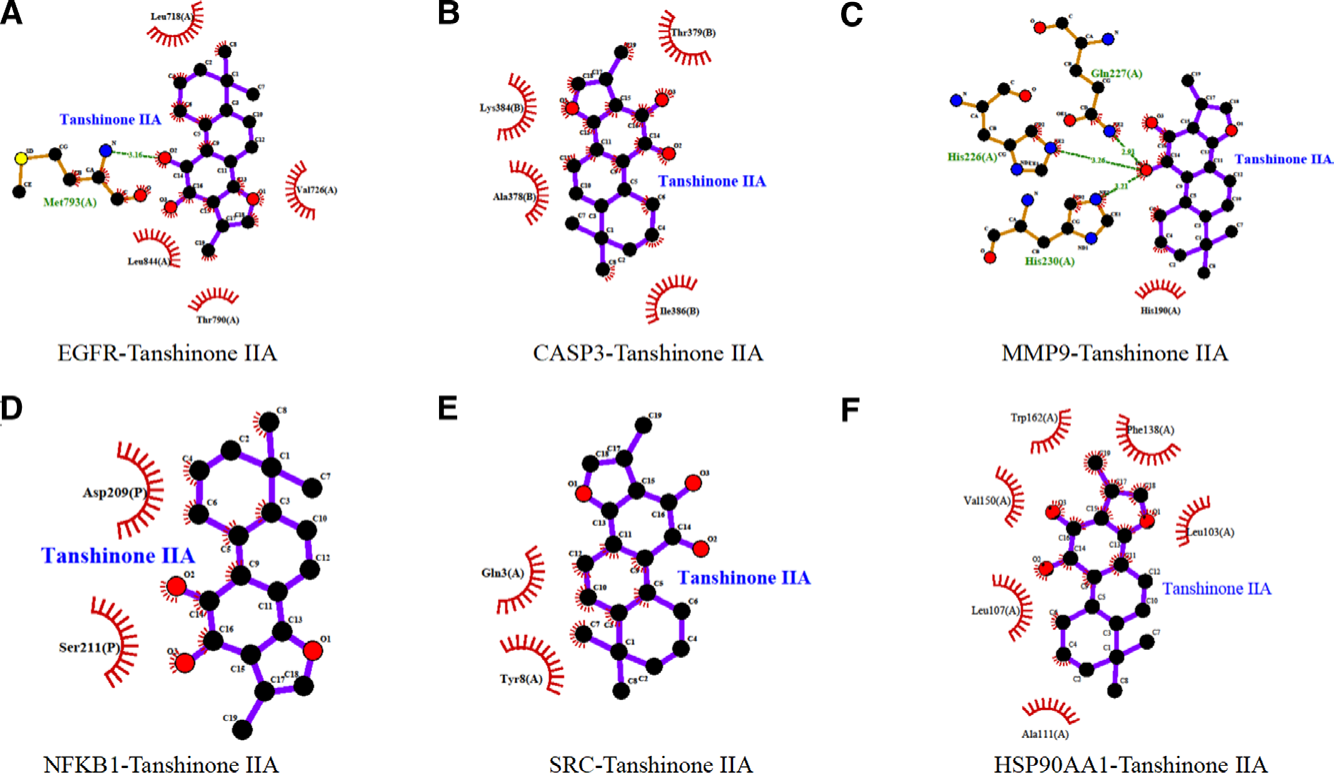
2D representation of the docking of Tanshinone IIA with protein receptors.

Tanshinone IIA was molecularly docked to the 6 core targets using Autodock Vina software, if the binding energy was ≤ −5.0 kcal/mol it indicated a better binding ability, the lower the binding energy, i.e. the more stable the binding. The results showed that the binding energy of tanshinone IIA were −9.0 kcal/mol with EGFR, −7.0 kcal/mol with CASP3, −8.2 kcal/mol with MMP9, −7.7 kcal/mol with NFKB1, −6.4 kcal/mol with SRC, and −10.4 kcal/mol with HSP90AA1 with a binding energy of −10.4 kcal/mol. PyMOL software was used to show 3D plots of molecular docking results of active ingredients with proteins, as shown in Figures 8 and 9. 2D plots of molecular docking results of active ingredients with proteins were drawn using LigPlot software, as shown in Figure 10.

## 4. Discussion

COPD is a respiratory disease that poses a serious risk to human health, with pathological changes involving damage to the lung parenchyma and small airways, leading to chronic airway obstruction, increased respiratory resistance, and pulmonary insufficiency. COPD was prevalent among smokers and individuals aged 50 and above. With the increasing smoking rates in developing countries and the aging population in high-income countries, the prevalence of COPD is expected to continue rising. It is projected that by 2060, approximately 5.4 million people will die annually from COPD and its complications such as pulmonary hypertension, recurrent pneumonia, and respiratory failure.^[11]^ Currently, there was no cure for COPD, and clinical treatment mainly focuses on symptom relief. However, this approach often failed to meet the treatment needs of patients, as traditional inhalation formulations are not sufficient to prevent acute exacerbations of COPD. Therefore, there is an urgent need to develop new drugs and disease targets to provide novel treatment strategies for COPD. Recent studies have shown that tanshinone IIA exhibited a positive role in treating COPD and its complications.^[12]^ Nevertheless, we still lack a clear understanding of the specific mechanisms of tanshinone treatment of COPD. Currently, network pharmacology and molecular docking techniques were widely applied in the screening of key targets for compound therapy and docking, providing possibilities for the initial exploration of the therapeutic mechanism of tanshinone IIA in treating COPD.

This study obtained 104 relevant targets for tanshinone IIA in treating COPD through network pharmacology and molecular docking methods. Among them, genes such as EGFR, CASP3, MMP9, NFKB1, SRC, and HSP90AA1 were identified as core targets. Currently, research on the EGFR signaling pathway was widespread, especially in its significant role in lung development, homeostasis, and repair, and drugs targeting targeting the EGFR signaling pathway may serve as novel therapies for treating COPD.^[13]^ Studies have shown that activation of the intrinsic protein kinase of EGFR upon ligand binding regulates the PI3K-AKT signaling pathway, leading to excessive airway mucus secretion and exacerbating the condition in COPD patients.^[14]^ An imbalance of proteases and antiproteases is an important pathogenesis of COPD, and a number of protein hydrolyzing enzymes in the body can destroy the structure of normal lung tissue. Among them, CASP3 and MMP9 belong to one of the classes of cysteine proteases and matrix metalloproteinases, respectively, which are able to degrade all components of the extracellular matrix (ECM), leading to structural abnormalities of the airway wall, and are closely associated with lung diseases, such as COPD, pulmonary fibrosis and lung cancer.^[15]^ The NFKB1 (p105/p50) subunit was an active regulatory factor of the NF-κB family genes, playing a crucial role in the inflammatory response in COPD. A study conducted on 180 healthy controls and 186 patients with acute exacerbations of COPD examined fasting peripheral blood. It was found that in patients with acute exacerbations of COPD, the expression levels of NFKB1 mRNA as well as levels of inflammatory factors such as IL-1β, IL-6, IL-8, IL-12, and TNF-α were elevated in peripheral blood.^[16]^ SRC was a type of non-receptor tyrosine kinase that can serve as a target for preventing and treating smoking-related lung injuries, such as COPD, and lung cancer.^[17]^ Targeting SRC may provide a new therapeutic approach for corticosteroid-insensitive lung disease, as chemical inhibitors of SRC (saracatinib and dasanitib) enhance the expression of the cellular antioxidant glutathione peroxidase-1 to prevent cigarette smoke-induced lung inflammation and tissue destruction.^[18]^ HSP90AA1 (HSP90α) is an isoform of heat shock proteins (HSP90) and a potential target for inducing the progression of COPD to squamous cell lung cancer.^[19]^ The airway goblet cell metaplasia was a hallmark of disability in COPD, and sustained airway goblet cell metaplasia requires HSP90 activity, which could be restored and disease progression slowed with the use of an HSP90 inhibitor, such as geldanamycin.^[20]^ From this, it was evident that EGFR, CASP3, MMP9, NFKB1, SRC, and HSP90AA1 played important roles in the occurrence and development of COPD. Targeted inhibition of these genes could provide new strategies and methods for the treatment of COPD.

Through GO enrichment and KEGG pathways analysis, it was found that the targets of tanshinone IIA may improve COPD by regulating responses to response to lipopolysaccharide, positive regulation of MAPK cascade and positive regulation of kinase activity. These enrichments were related to response to lipopolysaccharide, response to molecule of bacterial origin, positive regulation of cytokine production, positive regulation of MAPK cascade, positive regulation of kinase activity, PI3K-Akt signaling pathway, and relaxin signaling pathway. The PI3K/Akt signaling pathway is involved in a variety of physiological processes and serves as a pivotal regulator of the inflammatory response and oxidative stress in COPD, thus playing a pivotal role in both the progression and treatment of the disease.^[21]^ EGFR, as an upstream of the PI3K/Akt signaling pathway, could exacerbate COPD pathology by activating the EGFR/PI3K/AKT pathway to up-regulate the expression of inflammatory factors and induce inflammation in the lungs.^[22]^ SRC was a central hub of multiple signaling pathways and mediated the PI3K/AKt pathway to influence mitosis and tumor cell apoptosis.^[23]^ NFKB1, CASP3 and HSP90AA1 are downstream of the PI3K/Akt signaling pathway, and the PI3K/Akt pathway may regulate NF-κB and caspase-3-mediated apoptosis in lung cells, which affects lung function and histomorphology in lung-injured rats and mice.^[24,25]^ PI3K/AKT pathway modulated HSP90AA1 involved in reversing small cell lung cancer (SCLC) resistance to chemotherapy/radiotherapy treatment.^[26]^ Airway/lung remodeling and fibrosis were central features of inflammatory lung diseases such as COPD, asthma and pulmonary fibrosis.^[27]^ Relaxin/glucagon-like family peptide receptor 1 was the only relaxin receptor known to be expressed in the lung, and its expression level decreased when airway/lung remodeling and fibrosis occur.^[28]^ Relaxin not only affected airway/lung remodeling and fibrosis by modulating collagen deposition in the airways and lungs but also exerted anti-inflammatory effects by reducing the influx of neutrophils and mast cells into damaged organs or by inhibiting mast cell release.^[29]^ This showed that the PI3K-Akt signaling pathway and the relaxin signaling pathway played important roles in the treatment of COPD with tanshinone IIA.

Molecular docking showed that the binding energies of tanshinone IIA and the core targets were all less than ≤ −5.0 kcal/mol, demonstrating good affinity. Tanshinone IIA is an important characteristic component and active ingredient of Danshen, which can regulate EGFR, NF-κB, Caspase-3, and TNF-α proteins, as well as MAPK signaling pathway, PI3K/Akt signaling pathway, and TGF-β/Smad signaling pathway, and directly or indirectly affect respiratory system diseases.^[30]^ Tanshinone IIA was found to disrupt EGFR-Akt signaling, thereby inhibiting the occurrence of non-small cell lung cancer tumors.^[31]^ Tanshinone IIA was able to influence lung inflammation, oxidative stress, and cell apoptosis by inhibiting the expression of Caspase-3, as well as reducing the infiltration of CD44 and CD163 positive inflammatory cells, along with the expression of inflammatory cytokines such as IL-1β, TNF-α, and IL-6, through the suppression of the PI3K/Akt signaling pathway.^[32]^ Tanshinone IIA attenuated pathological lung injury, apoptosis, neutrophil infiltration, pulmonary edema and inflammatory response by inhibiting NF-κB pathway activation in the lungs of rats with sepsis lung injury model constructed by cecum ligation and puncture.^[33]^ Tanshinone IIA inhibited proliferation and migration by downregulating the PI3K/Akt pathway in non-small cell lung cancer.^[34]^ STS attenuates lung function, mucus secretion, bronchodilatation, bronchial collagen deposition, inflammatory response and oxidative stress in mice with acute exacerbation of COPD induced by cigarette smoke and LPS exposure via inhibition of NF-κB activation.^[35]^ STS inhibited the activation of the MAPK/HIF-1α signaling pathway to reduce the production of TNF-α, IL-1β, ROS, and MMP9, and affects the infiltration of inflammatory cells in the lungs of cigarette smoke-exposed mice, thereby improving lung function and emphysema.^[36]^ Tanshinone IIA inhibited SRC-mediated osteosarcoma progression downstream of PI3K/AKt and MAPK/ERK signaling pathways.^[23]^ In the prevention and treatment of ovarian cancer, tanshinone IIA, cryptotanshinone, and resveratrol regulated the PI3K-Akt signaling pathway by mediating the expression of HSP90AA1, CDK2, and PIK3CG, exerting anticancer effects.^[37]^ Based on the above findings, tanshinone IIA modulates core targets such as EGFR, CASP3, MMP9, NFKB1, SRC, and HSP90AA1 in the treatment of respiratory diseases and cancer.

In summary, this study explored and analyzed the mechanism of tanshinone IIA in the treatment of COPD using network pharmacology and molecular docking techniques. The results showed that tanshinone IIA played a key role in the prevention and treatment of COPD through the core targets of EGFR, CASP3, MMP9, NFKB1, SRC, and HSP90AA1. Similarly, this study provided new insights and methods for the prevention and treatment of COPD, which is of significant importance for exploring further pharmacological effects of tanshinone IIA.

## Author contributions

**Data curation:** Shili Yang, Xinyan Zhang.

**Formal analysis:** Huaiquan Liu, Xinyan Zhang.

**Funding acquisition:** Bo Chen.

**Project administration:** Bo Chen.

**Software:** Shili Yang, Shuoshuo Shao.

**Validation:** Shuoshuo Shao.

**Writing – original draft:** Huaiquan Liu.
